# Nesfatin-1 decreases the motivational and rewarding value of food

**DOI:** 10.1038/s41386-020-0682-3

**Published:** 2020-04-30

**Authors:** Riccardo Dore, Regina Krotenko, Jan Philipp Reising, Luca Murru, Sivaraj Mohana Sundaram, Alessandro Di Spiezio, Helge Müller-Fielitz, Markus Schwaninger, Olaf Jöhren, Jens Mittag, Maria Passafaro, Marya Shanabrough, Tamas L. Horvath, Carla Schulz, Hendrik Lehnert

**Affiliations:** 1 Department of Internal Medicine I, University of Lübeck, Ratzeburger Allee 160, 23562 Lübeck, Germany; 2 Center of Brain, Behavior and Metabolism (CBBM), University of Lübeck, Ratzeburger Allee 160, 23562 Lübeck, Germany; 3 CNR, Institute of Neuroscience, 20129 Milan, Italy; 4 Institute for Experimental and Clinical Pharmacology and Toxicology, University of Lübeck, Ratzeburger Allee 160, 23562 Lübeck, Germany; 5 Department of Comparative Medicine, Program on Integrative Cell Signaling and Neurobiology of Metabolism, Yale University School of Medicine, New Haven, CT 06520 USA; 6 Department of Anatomy and Histology, University of Veterinary Medicine, Budapest, H-1078 Hungary; 7 Present Address: Department of Women’s and Children’s Health, Karolinska Institutet, 171 76 Stockholm, Sweden; 8 Present Address: Department of Biochemistry, University of Kiel, 24118 Kiel, Germany

**Keywords:** Motivation, Reward

## Abstract

Homeostatic and hedonic pathways distinctly interact to control food intake. Dysregulations of circuitries controlling hedonic feeding may disrupt homeostatic mechanisms and lead to eating disorders. The anorexigenic peptides nucleobindin-2 (NUCB2)/nesfatin-1 may be involved in the interaction of these pathways. The endogenous levels of this peptide are regulated by the feeding state, with reduced levels following fasting and normalized by refeeding. The fasting state is associated with biochemical and behavioral adaptations ultimately leading to enhanced sensitization of reward circuitries towards food reward. Although NUCB2/nesfatin-1 is expressed in reward-related brain areas, its role in regulating motivation and preference for nutrients has not yet been investigated. We here report that both dopamine and GABA neurons express NUCB2/nesfatin-1 in the VTA. Ex vivo electrophysiological recordings show that nesfatin-1 hyperpolarizes dopamine, but not GABA, neurons of the VTA by inducing an outward potassium current. In vivo, central administration of nesfatin-1 reduces motivation for food reward in a high-effort condition, sucrose intake and preference. We next adopted a 2-bottle choice procedure, whereby the reward value of sucrose was compared with that of a reference stimulus (sucralose + optogenetic stimulation of VTA dopamine neurons) and found that nesfatin-1 fully abolishes the fasting-induced increase in the reward value of sucrose. These findings indicate that nesfatin-1 reduces energy intake by negatively modulating dopaminergic neuron activity and, in turn, hedonic aspects of food intake. Since nesfatin-1´s actions are preserved in conditions of leptin resistance, the present findings render the NUCB2/nesfatin-1 system an appealing target for the development of novel therapeutical treatments towards obesity.

## Introduction

Nesfatin-1 is a cleavage product of nucleobindin-2 (NUCB2) whose sequence is highly conserved from fish to mammals [1]. The anorexigenic properties of nesfatin-1 were reported for the first time by Oh-I and collaborators [2]. This seminal finding was confirmed and further extended by other research groups [3–5] and by us [6, 7]. In addition, nesfatin-1 administration into the central nervous system (CNS) was also shown to increase body core temperature [8] and energy expenditure [6, 7, 9], thereby indicating its major role in the central regulation of energy homeostasis. In fact, NUCB2 and/or nesfatin-1 (here named NUCB2/nesfatin-1 as common antibodies do not distinguish between NUCB2 and nesfatin-1) is highly expressed in homeostatic centers such as the hypothalamus and brainstem [2, 10–13]. Moreover, it is also found in a number of peripheral tissues, including the pancreatic beta cells [14, 15], adipose depots [16, 17] and gastric mucosa, which is considered the primary source of NUCB2/nesfatin-1 as its expression is ~10-fold higher than in the brain [18]. Thus, NUCB2/nesfatin-1 can be released into the bloodstream, crossing the blood-brain-barrier by a non-saturable mechanism [19, 20] and reach the CNS.

While homeostatic feeding is mainly driven by the need to maintain biological functions, hedonic regulation of feeding is based on the voluntary consumption of food potentially exceeding the energy demand. Brain dopamine neurons, especially those originating in the ventral tegmental area (VTA) and projecting to the nucleus accumbens (NAcc), were previously shown to be essential for homeostatic functions such as drinking and chow feeding [21], and to play a pivotal role in the formation of reward-related processes such as the regulation of motivational properties and reward value of energy-dense food [22]. Hedonic signals can weaken or even disrupt satiety mechanisms controlled by homeostatic pathways and may potentially lead to eating disorders such as binge eating disorder, obesity and food addiction [23, 24]. Thus, a tight regulation of reward system’s functions and its coupling to homeostatic needs is of crucial importance.

While there is ample data on nesfatin-1´s role in the homeostatic control of feeding and energy balance, several evidence also suggests that nesfatin-1 can act on the reward system to affect hedonic feeding-related behaviors. NUCB2/nesfatin-1 is expressed in a number of reward-related brain regions such as the ventral and dorsal striatum, lateral hypothalamus, dorsal and dorsolateral tegmental nucleus [13]. Moreover, nesfatin-1 decreases chow food intake when administered bilaterally into the VTA and dopamine release in the NAcc when administered intracerebroventricularly [25]. Also, nesfatin-1 hyperpolarizes dopamine neurons and reduces dose-dependently their firing rate in the substantia nigra (SN) and VTA [25, 26].

Taken together, the NUCB2/nesfatin-1 system may act within reward circuitries to modulate aspects of food reward. To investigate this in detail, we first determined NUCB2/nesfatin-1 co-localization with tyrosine hydroxylase (TH), glutamate decarboxylase 67 (GAD67) and calretinin (as markers for dopamine and GABA neurons) in the VTA. Next, to examine whether nesfatin-1 directly affects the activity of dopamine and/or GABA neurons of the VTA through the activation of potassium channels, ex vivo whole-cell patch clamp recordings were performed. Additionally, to measure willingness to work for sucrose under low- and high-effort conditions, mice were tested in the fixed ratio 1 and progressive ratio schedule of reinforcement upon intracerebroventricular (i.c.v.) or VTA-specific nesfatin-1 administration. Finally, to assess whether nesfatin-1 modulates the reward value of sucrose, mice were tested in a 2-bottle choice procedure coupled to optogenetic stimulation of VTA dopamine neurons [27–29] under fasting conditions (hence, with presumably low endogenous levels of NUCB2/nesfatin-1 mRNA and/or protein) and after i.c.v. nesfatin-1 administration.

## Materials and methods

### Mice

For in vivo studies, male C57BL/6 J mice (Charles River) were single housed in wire-topped, plastic cages with bedding and nesting material, in a 12 h/12 h light/dark cycle (lights off at 9:00 am) temperature-controlled (21 °C) air flow cabinet. Mice had *ad libitum* access to corn-based chow (#1320; Altromin, Germany) and water, unless otherwise stated. For in vivo optogenetic studies, dopamine transporter (DAT)-Cre mice were employed, whereas in vitro validation of the optogenetics was performed in DAT-Cre-ZsGreen mice. The experimental protocols for animals and their care were in accordance with the directive 2010/63/EU of the European Parliament and were approved by the committee on animal care of the state of Schleswig-Holstein, Germany. The ‘PHS Policy on Humane Care and Use of Laboratory Animals’ (NIH publication no. 15-8013, revised 2015) were followed.

### Double-fluorescence Immunohistochemistry

Double-fluorescence immunohistochemistry was performed on coronally sectioned 4% paraformaldehyde (PFA) fixed brain tissue from wild-type *ad libitum* fed mice. To detect immunofluorescence, sections (40 μm) were incubated with the following antibodies (NUCB2/nesfatin-1: #H-003-22, 1:1000, Phoenix Pharmaceuticals; tyrosine hydroxylase: #T1299, 1:500, Sigma Aldrich; calretinin: #AB1550, 1:1000, Millipore; GAD67: #MAB5406, 1:1000, Millipore) followed by species-specific Alexa 488, 633 and 647 secondary antibodies. Fluorescence images were acquired on a Leica SP5 confocal microscope and analyzed with Image J software (NIH). For details, see Supplementary Materials and Methods.

### Ex vivo electrophysiology

Horizontal brain slices containing the VTA were prepared from C57BL/6 J mice brain following standard procedures with minor modifications [30]. Slices were transferred to a recording chamber and superfused with artificial cerebrospinal fluid (aCSF) at a rate of ~2 ml/min at 33 °C. Patch-clamp electrodes were filled with a K-gluconate-based internal solution. Slices were superfused with aCSF supplemented with kynurenic acid (3 mM) and bicuculline (20 μM), and VTA dopamine and GABA neurons were clamped at a holding potential of −50 mV. Slices were then perfused with nesfatin-1 (10 nM) [25, 26] followed by the potassium channels inhibitor BaCl_2_ (1 mM). Experiments were analyzed offline with Axon Clampfit 10.1 software (Molecular Devices, US). For details, see Supplementary Materials and Methods.

### Laser capture microdissection (LCM) and qRT-PCR

Mice were sacrificed by cervical dislocation, brains were dissected and placed in dry ice. Coronal brain slices (20 μm) containing the VTA or PVN were collected on slides and stored at -80 °C. Subsequently, brain regions were identified under the microscope, laser-cut (CryLaS, Germany; Fig. S1a, b) and collected in plastic vials. For each animal, the VTA or PVN from 3 consecutive slices were pooled, lysis buffer and beta-mercaptoethanol were added immediately after, and samples were stored at −80 °C. Extraction of the total RNA and synthesis of the first-strand cDNA were performed as previously described [31]. Messenger RNA levels were determined by qRT-PCR as reported earlier [32]. The specificity of qRT-PCR amplification was verified by analysis of melting curves (Fig. S1d). Oligonucleotide primers were obtained from Invitrogen, US (Fig. S1e).

### Intracranial surgery and microinjection procedures

Mice underwent unilateral implantation of a 26-gauge stainless steel cannula (PlasticsOne, US) under stereotaxic control (Kopf Instruments, US). The following coordinates were used (relative to bregma, in mm): lateral ventricle, AP: −0.22, ML: ± 1.00, DV: −1.50 from the skull surface; VTA, AP: −3.40, ML: ±0.35, DV: −3.80 from the skull surface. Incisor bar was set at −2.00 mm below the interaural line, according to Paxinos and Franklin [33]. For details, see Supplementary Materials and Methods.

### Pharmacological treatments

Human recombinant nesfatin-1 (Sigma-Aldrich; US) was dissolved in phosphate-buffered saline (PBS) or aCSF. Aliquots were kept at −80 °C and working solutions were prepared on the experimental day and kept on blue ice.

### Food intake assessment

At the beginning of the dark phase, mice were i.c.v. injected in a counterbalanced experimental design with nesfatin-1 (100 and 300 pmol) [34] or PBS. Given the long duration of the measurements as well as to match the experimental conditions of previous studies [3, 34], food was provided immediately after drug administration and the intake was determined 1.5 and 3.5 h later to the nearest 0.01 g.

### Fixed and progressive ratio schedule of reinforcement for sucrose

After training the mice under the fixed ratio 1 and 3 schedule of reinforcement, the progressive ratio schedule of reinforcement was introduced, in which the number of active lever presses required to obtain successive rewards increased within the session. Sessions ended either after 60 min or prematurely if mice had not pressed the active lever for at least 10 min, whichever came first. Experimental testing began upon reaching stable performance (<15% variation in the breakpoint across 3 consecutive days). On testing day, to minimize any handling-related impact on the animals´ performance, mice were administered with nesfatin-1 (i.c.v.: 100 and 300 pmol; VTA-specific: 50 pmol) or PBS 30 min prior to behavioral sessions. This pretreatment time was chosen to allow diffusion of the drugs, and to minimize the effects of handling and injection procedures on the animals´ performance. In a control experiment, mice undergoing the fixed ratio 1 schedule of reinforcement were administered with nesfatin-1 (i.c.v.: 300 pmol; VTA-specific: 50 pmol) or PBS. The experiments were performed in a counterbalanced design and with at least 3 days of washout period. For details, see Supplementary Materials and Methods.

### Long-lasting 2-bottle choice procedure

Mice were presented daily with a 87 mM (~3%) sucrose solution with food and water freely available in their home cage for 4 h/day (9:00 am – 1:00 pm) for one week. To account for side preference, the bottles of sucrose and water were swapped daily. Upon reaching stable preference for the sucrose solution (<5% variation across 3 consecutive days), mice received an i.c.v. administration of nesfatin-1 (300 pmol) or PBS in a counterbalanced experimental design. Given the long duration of the measurements as well as to match the experimental conditions of the food intake study, bottles were presented to the animals immediately after drug administration. The preference for the sucrose solution was calculated as follows: sucrose solution intake (g) / total solutions intake (g) × 100.

### Virus injection and fiber optic implantation

To achieve dopamine neuron-specific expression of the photosensitive ion channel Channelrhodopsin-2 (ChR2), DAT-Cre mice were stereotaxically injected unilaterally with 1 µl of purified Cre recombinase-dependent rAAV-FLEX-*rev*-ChR2-TdTomato (ChR2^+^) or rAAV-FLEX-*rev*-TdTomato vector (ChR2^-^) [35] into the VTA (relative to bregma, in mm: AP −3.40; ML −0.35; DV −4.20 from the skull surface). A fiber optic (Thorlabs, US) was then implanted above the VTA (DV −4.00 from the skull surface). Finally, a 26-gauge guide cannula was implanted aiming at the lateral ventricle as described above (Fig. S4a). For immunofluorescence in DAT-Cre-ZsGreen mice and details, see Supplementary Materials and Methods.

### Optogenetic setup

The optogenetic setup was similar to that previously described by Domingos et al. [27–29]. For details, see Supplementary Materials and Methods.

### Behavioral validation of optogenetics: 2-bottle choice and conditioning procedures

To assess the reinforcing efficacy of the optogenetic activation of VTA dopamine neurons, we employed the 2-bottle choice and conditioning procedures. A positive response to these two behavioral procedures was used as inclusion criteria for the subsequent behavioral tests. For details, see Supplementary Materials and Methods.

### Validation of the short-lasting 2-bottle choice procedure employing optogenetics

After the behavioral validation of the optogenetic technique, the configuration of the 2-bottle choice procedure was progressively changed over 4 days, with *ad libitum* fed ChR2^+^ mice undergoing daily 20-min behavioral session during the second half of the dark phase (from 3 to 6 pm), to allow mice to eat for at least 6 h and thus to have a physiologically satiated state. On day 1, mice had access to sucrose (87 mM; ~3%) against water + laser OFF. Since mice previously underwent a conditioning procedure, sucrose bottle on day 1 was placed in the opposite side to which mice were conditioned. Next, mice had access to sucrose against water + laser ON on day 2, to sucrose against sucralose (0.5 mM) + laser OFF on day 3 and to sucrose against sucralose + laser ON on day 4 (Fig. 5a). The position of the laser bottle was the same across days as mice were expected to spontaneously shift their preference due to experimental manipulations (laser ON/OFF and water/sucralose). Mice were always tethered to the rotary joint patch cable to prevent reward expectation. Solutions were prepared daily with tap water.

### Short-lasting 2-bottle choice procedure employing optogenetics

To test the effects of nesfatin-1 on the reward value of sucrose, we used the configuration sucrose against sucralose + laser ON, with the latter functioning as reference stimulus [27]. Behavioral sessions were performed in the second half of the dark phase (3:00pm–6:00pm) and lasted 20 min. The position of the laser bottle was the same across days as mice were expected to spontaneously shift their preference daily due to pharmacological and fasting procedures. Chow food was not present during the behavioral sessions. On testing day, mice under different metabolic conditions (*ad libitum* and 6–8 h fasting) were administered i.c.v. with nesfatin-1 (300 pmol) or PBS 30 min prior to behavioral sessions. This pretreatment time was chosen to allow diffusion of the drugs, and to minimize the effects of handling and injection procedures on the animals´ performance.

### Statistical analysis

All statistical analyses were performed in GraphPad Prism 7.02 (GraphPad, US). Data was analyzed using either one-way or two-way repeated measures analysis of variance (RM-ANOVA), followed by Tukey’s or Dunnett´s *post hoc* tests, or Student´s *t* test, where appropriate. Significance was set at *P* < 0.05. All data are expressed as mean ± standard error of the mean (SEM). Experimental designs and samples sizes aimed at minimizing usage and distress of animals, and were sufficient for detecting robust effect sizes. For details, see Supplementary Materials and Methods.

## Results

### NUCB2/nesfatin-1 is expressed in the VTA and dopamine neurons respond to nesfatin-1

First, NUCB2/nesfatin-1 mRNA levels in the VTA were quantified and compared with those in the PVN, a brain site where this peptide is heavily expressed [2]. NUCB2/nesfatin-1 mRNA was detected in all VTA samples, with a ~55% lower expression than in the PVN (Fig. S1c, d).

**Fig. 1:**
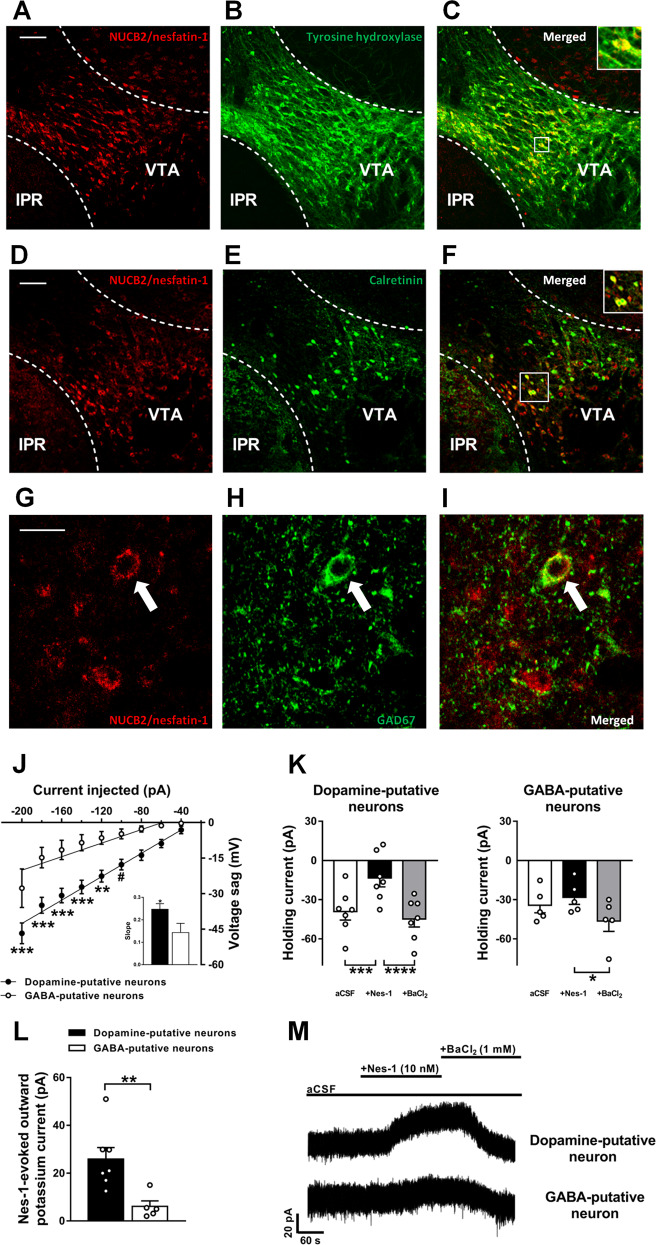
NUCB2/nesfatin-1 is expressed in the VTA and hyperpolarizes dopamine, but not GABA, neurons. Representative confocal images of wild-type mice midbrain coronal section (bregma: −3.40 mm) showing NUCB2/nesfatin-1 (**a** and **d**), TH (**b**) and calretinin (**e**) positive neurons in the VTA, and merged images showing that NUCB2/nesfatin-1-containing neurons also express TH (**c**) and calretinin (**f**), markers for dopamine and GABA neurons, respectively. Confocal images of NUCB2/nesfatin-1 (**g**) and GAD67 (**h**) positive neurons in the VTA, and merged image showing one representative NUCB2/nesfatin-1-containing neuron also expressing GAD67 (**i**). Scale bar: 100 µm in A-F and 20 µm in G-I. IPR, rostral interpeduncular nucleus; VTA, ventral tegmental area. Putative dopamine and GABA neurons of the VTA were identified electrophysiologically by sag potential amplitude in response to negative current injection (−20 pA steps) (**j**). Inset shows the comparison of the slopes of the linear regression lines. Mean quantification of the baseline (aCSF) holding current shift induced by nesfatin-1 and BaCl_2_ superfusion of putative dopamine and GABA neurons of the VTA (K) (*N*_neurons_ = 5–7, *N*_mice_ = 4). Mean quantification of nesfatin-1-evoked potassium outward current in putative dopamine and GABA neurons of the VTA (**l**). Representative holding current traces from VTA neurons held at -50 mV of wild-type mice showing the outward current evoked by bath application of nesfatin-1 (10 nM) in putative dopamine neurons, which was blocked by BaCl_2_ (1 mM). No effects in putative GABA neurons were found (M). Data represent mean ± SEM. ^#^*P* < 0.1, *P < 0.05, **P < 0.01, ***P < 0.001 and ****P < 0.0001 vs. respective control group (Tukey’s *post hoc* test or Student’s *t* test).

Double-fluorescence immunohistochemistry confirmed the presence of NUCB2/nesfatin-1-expressing neurons in the VTA. Specifically, we found that 60.7 ± 6.0% of NUCB2/nesfatin-1 neurons expressed TH and that 37.2 ± 6.0% of TH neurons expressed NUCB2/nesfatin-1 (Fig. 1a–c; *N* = 5). Moreover, 35.0 ± 0.7% of NUCB2/nesfatin-1 neurons expressed calretinin and 52.9 ± 6.5% of calretinin neurons expressed NUCB2/nesfatin-1 (Fig. 1d–f; *N* = 5). The expression of NUCB2/nesfatin-1 in VTA GABA neurons was further confirmed by co-staining of NUCB2/nesfatin-1 and GAD67 (Fig. 1g–i). In absolute terms, considering that the TH-expressing neurons are much more numerous than calretinin-expressing neurons (~282 vs. ~113 neurons/section, respectively), these results suggest that dopamine neurons may be the primary source of nesfatin-1 in the VTA.

Additionally, we performed whole-cell patch clamp recordings from VTA dopamine neurons, to confirm the nesfatin-1-induced hyperpolarizing effects [25, 26], as well as from VTA GABA neurons. Typically, VTA dopamine neurons are identified by the presence of long action potential duration, hyperpolarization activated cation current (*I*_h_), low spontaneous firing frequency and depolarized resting membrane potential [36]. However, it was reported that VTA dopamine and GABA neurons can display similar spontaneous *I*_h_ current and firing activity [37, 38]. More recently, the identification of VTA dopamine and GABA neurons by *I*_h_-related sag potential amplitude and rebound spiking activity in response to hyperpolarization was clearly confirmed by molecular and genetic profiling of the neurons [39]. Here, similarly to Merrill and colleagues’ study [39], we found that sag potential amplitude increased with increasing negative current injection in a linear and neuronal type-specific manner (neuron type: *F*[1,11] = 14.49, *P* < 0.01; current: *F*[8,88] = 59.63, *P* < 0.0001; neuron type × current: *F*[8,88] = 4.54, *P* < 0.001; Figs. 1j and S2). A significant effect of treatment was observed in both putative dopamine (*F*[2, 12] = 27.81, *P* < 0.0001) and GABA neurons (*F*[2,8] = 7.09, *P* < 0.01; Fig. 1k, m). However, *post-hoc* analysis revealed that only in putative dopamine neurons there was a significant effect of nesfatin-1 treatment on the holding current (*P* < 0.001), which was fully blocked by BaCl_2_ application (*P* < 0.0001). Instead, in putative GABA neurons, there was no effect of nesfatin-1 treatment (*P* = 0.463) but only a hyperpolarizing effect induced by BaCl_2_ application (*P* < 0.05; Fig. 1k, m). Overall, nesfatin-1 application elicited a larger outward current in dopamine neurons which was significantly different from that elicited in GABA neurons of the VTA (*t*[10] = 3.24, *P* < 0.001; Fig. 1l). These data indicate that nesfatin-1 hyperpolarizes dopamine neurons *via* a direct mechanism independent of GABA neurotransmission, and therefore suggest that a nesfatin-1 putative receptor is expressed by dopamine neurons.

### Central nesfatin-1 reduces motivation to work for sucrose

**Fig. 2:**
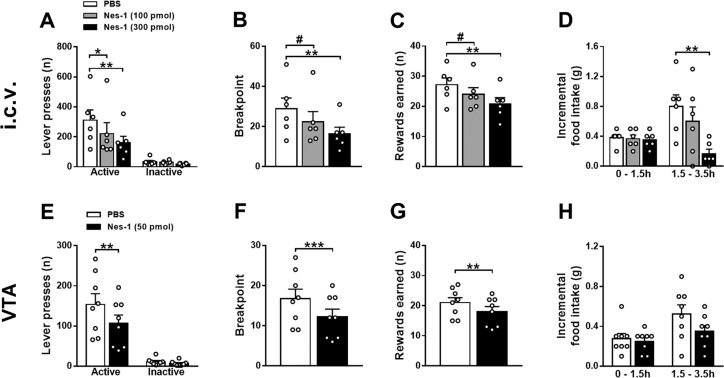
Central nesfatin-1 reduces motivation to work for sucrose in a high-effort condition. Effects of i.c.v. administration of nesfatin-1 on the number of lever presses (**a**), breakpoint (**b**), rewards earned (**c**) and home cage chow food intake (**d**) in *ad libitum* fed mice in the progressive ratio schedule of reinforcement (*N* = 6). Effects of VTA-specific administration of nesfatin-1 on the number of lever presses (**e**), breakpoint (**f**), rewards earned (**g**) and home cage chow food intake (H) in *ad libitum* fed mice (*N* = 8) in the progressive ratio schedule of reinforcement. Operant behavioral sessions were performed at the beginning of the dark phase, began 30 min after drug administration and lasted 1 h. Data represent mean ± SEM. *P < 0.05, **P < 0.01 and ***P < 0.001 vs. PBS group (Dunnett´s *post hoc* test or paired Student’s *t* test).

Given the responsiveness of VTA dopamine neurons to nesfatin-1 ex vivo, we tested whether central nesfatin-1 could affect motivated behavior by employing the progressive ratio schedule of reinforcement. As shown in Figs. 2a–c and S3a, mice administered with i.c.v. nesfatin-1 dose-dependently worked less to obtain sucrose. Treatment with nesfatin-1 affected the number of lever presses (treatment: *F*[2,10] = 10.86, *P* < 0.01; lever: *F*[1,5] = 11.93, *P* < 0.05; treatment × lever: *F*[2,10] = 8.29, *P* < 0.05; Fig. 2a). The active lever presses (*F*[2,10] = 9.99, *P* < 0.01; Fig. 2a), breakpoint (*F*[2,10] = 9.52, *P* < 0.01; Fig. 2b), rewards earned (*F*[2,10] = 9.97, *P* < 0.01; Fig. 2c) and session duration (*F*[2,10] = 7.70, *P* < 0.01; Fig. S3a) were all reduced after i.c.v. administration of nesfatin-1. The inactive lever presses (*F*[2,10] = 2.03, n.s.; Fig. 2a) as well as inactive lever presses rate (*F*[2,10] = 0.62, n.s.; Fig. S3b) were unaffected by the i.c.v. treatment with nesfatin-1. Additionally, a new cohort of mice (*N* = 6) was tested for locomotion 30 min after i.c.v. drug administration and results demonstrate that nesfatin-1 (100 and 300 pmol) did not affect the number of infrared beam breaks (*F*[2,10] = 2.57, n.s.; data not shown).

Additionally, the results in Figs. 2e–g and S3c show that also mice administered with nesfatin-1 into the VTA worked less to obtain sucrose. Treatment with nesfatin-1 affected the number of lever presses (treatment: *F*[1,7] = 25.58, *P* < 0.01; lever: *F*[1,7] = 26.80, *P* < 0.01; treatment × lever: *F*[1,7] = 26.21, *P* < 0.01; Fig. 2e). The active lever presses, breakpoint, rewards earned (Fig. 2e–g) and session duration (Fig. S3c) were all significantly reduced after VTA-specific administration of nesfatin-1. The inactive lever presses (Fig. 2e) as well as inactive lever presses rate (Fig. S3d) were unaffected by the VTA-specific treatment with nesfatin-1.

Treatment with i.c.v. nesfatin-1 affected home cage food intake (treatment: *F*[2,10] = 19.33, *P* < 0.001; time: *F*[1,5] = 1.14, n.s.; treatment × time: *F*[2,10] = 5.75, *P* < 0.05; Fig. 2d); in fact, food intake was significantly reduced 3.5 h following nesfatin-1 administration but importantly, as discussed below, not following 1.5 h.  VTA-specific administration of nesfatin-1 did not affect home cage food intake (treatment: *F*[1,7] = 2.60, n.s.; time: *F*[1,7] = 6.86, *P* < 0.05; treatment × time: *F*[1,7] = 1.97, n.s.; Fig. 2h).

**Fig. 3:**
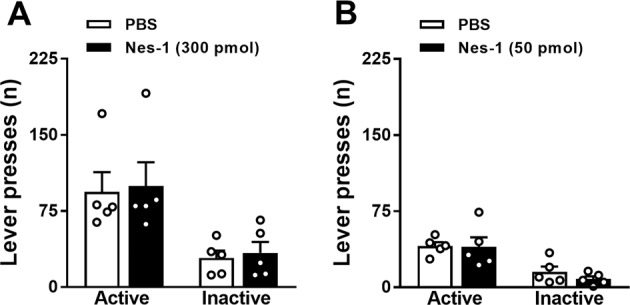
Central nesfatin-1 does not affect motivation to work for sucrose in a low-effort condition. Effects of nesfatin-1 on the number of lever presses upon either i.c.v. (**a**) or VTA-specific administration (**b**) in *ad libitum* fed mice in the fixed ratio 1 schedule of reinforcement (*N* = 5). Operant behavioral sessions were performed at the beginning of the dark phase, began 30 min after drug administration and lasted 1 h. Data represent mean ± SEM.

Furthermore, in two new cohorts of mice undergoing the fixed ratio 1 schedule of reinforcement, lever pressing was not affected by the treatment with nesfatin-1 either when injected i.c.v. (treatment: *F*[1,4] = 0.72, n.s.; lever: *F*[1,4] = 17.88, *P* < 0.05; treatment × lever: *F*[1,4] = 0.07, n.s.; Fig. 3a) or into the VTA (treatment: *F*[1,4] = 0.22, n.s.; lever: *F*[1,4] = 22.62, *P* < 0.01; treatment × lever: *F*[1,4] = 0.65, n.s.; Fig. 3b).

These data demonstrate that nesfatin-1 acts within the VTA to negatively modulate food motivation under high-effort conditions without affecting locomotion and homeostatic food intake.

### I.c.v. nesfatin-1 decreases sucrose preference in the long-lasting 2-bottle choice procedure

**Fig. 4:**
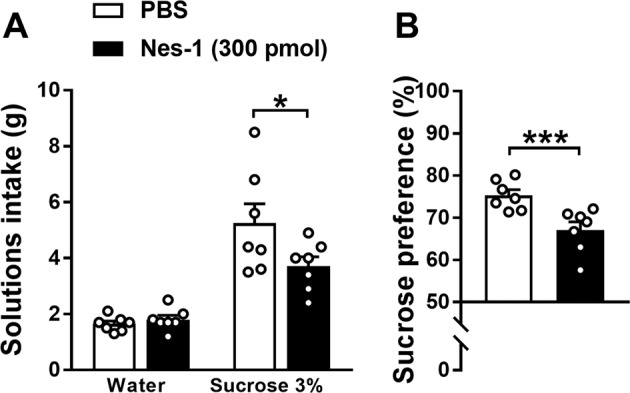
I.c.v. nesfatin-1 decreases sensitivity to rewarding food. Effects of i.c.v. administration of nesfatin-1 on solutions intake (**a**) and sucrose preference (**b**) assessed in a long-lasting 2-bottle choice procedure in *ad libitum* fed mice (*N* = 7). Behavioral sessions were performed in the first half of the dark phase, began immediately after drug administration and lasted 4 h. Mice were presented with two bottles immediately after drug administration. Data represent mean ± SEM. *P < 0.05 and ***P < 0.001 vs. PBS group (paired Student’s *t* test).

In addition, the long-lasting 2-bottle choice procedure was employed to assess sucrose preference upon i.c.v. drug administration. I.c.v. administration of nesfatin-1 significantly reduced sucrose intake (*t*[6] = 3.25, *P* < 0.05) whereas water intake was unaffected (*t*[6] = 1.06, n.s.; Fig. 4a). As a consequence, mice administered i.c.v. with nesfatin-1 displayed a significant reduction in the preference for sucrose (*t*[6] = 6.52, *P* < 0.001; Fig. 4b). These data indicate that nesfatin-1 modulates the sensitivity to rewarding food.

### Behavioral validation of optogenetics: 2-bottle choice and conditioning procedures

We next adopted the optogenetic technique for eliciting VTA dopamine neurons activation contingent with ingestion of liquids. By employing DAT-Cre mice, we achieved selective expression of ChR2 in VTA dopamine neurons, which was confirmed by immunohistochemistry (Fig. S4a, b). The reinforcing efficacy of VTA dopamine neurons optogenetic stimulation was confirmed behaviorally by employing the 2-bottle choice and conditioning procedures.

In the 2-bottle choice procedure (Fig. S4c), already on the first day, ChR2^+^ mice learned to discriminate between the laser bottle and the opposite water bottle (non-coupled to the laser). The performance of representative animals on day 1 is shown in Fig. S4d. On average, over 4 consecutive days of testing, the preference for the laser bottle was significantly higher in ChR2^+^ respect to ChR2^-^ mice (79.2% vs. 61.5%, respectively; *t*[12] = 3.48, *P* < 0.01; Fig. S4e).

In the conditioning procedure (Fig. S4f), we found that the effect of the laser stimulation on licking behavior was dependent on ChR2 expression in the VTA (laser: *F*[1,12] = 57.05, *P* < 0.00001; ChR2: *F*[1,12] = 11.34, *P* < 0.01; laser × ChR2: *F*[1,12] = 31.73, *P* < 0.001; Fig. S4g). While ChR2^+^ mice showed a significant ~10-fold increase in the number of licks at the laser bottle as compared with those at the water bottle non-coupled to the laser during the training phase (*P* < 0.001), in ChR2^-^ mice the number of licks was comparable (*P* = 0.597; Fig. S4g). Moreover, in the testing phase, during which the laser was kept OFF, ChR2^+^ mice displayed a higher preference for the bottle that was coupled to the laser during the training phase as compared with ChR2^-^ mice (73.1% vs. 47.6%; *t*[13] = 3.16, *P* < 0.01; Fig. S4h).

Even though it was recently shown that optogenetic stimulation in freely moving mice may affect neuronal activity and behavior despite the absence of opsin or fluorophore expression [40], our data from the ChR2^−^ control group demonstrate that the stimulation pattern employed here is neither reinforcing nor a strong visual cue. Therefore, since both ChR2 expression and lick-contingent optogenetic stimulation of VTA dopamine neurons were required to affect mice´ behavior, subsequent experiments were performed only in ChR2^+^ mice and “laser OFF” was used as a control condition.

### Validation of the short-lasting 2-bottle choice procedure employing optogenetics

**Fig. 5:**
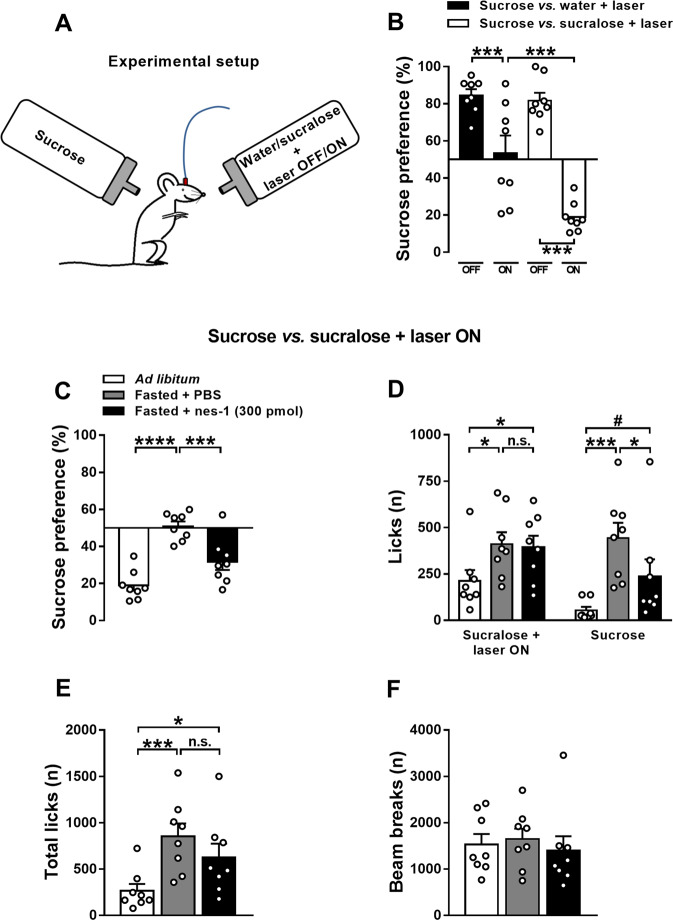
Validation of the short-lasting 2-bottle choice procedure employing optogenetics. Schematic drawing depicting the configuration of the 2-bottle choice procedure, with *ad libitum* fed mice receiving 1-sec-long pulse every 3 licks at the water or sucralose bottle (**a**). On day 1 (sucrose against water + laser OFF), mice showed a high preference for sucrose (84.6%; *t*[7] = 10.50, *P* < 0.0001; one-sample Student’s *t* test). The following day (sucrose against water + laser ON), mice showed a significant drop of the sucrose preference. On day 3 (sucrose against sucralose + laser OFF), mice displayed a high preference for sucrose (81.7%; *t*[7] = 7.57, *P* < 0.001; one-sample Student’s *t* test). The following day (sucrose against sucralose + laser ON), sucrose preference was completely reverted, with mice (*N* = 8) significantly preferring the sucralose + laser ON bottle (19.0%; *t*[7] = −10.97, *P* < 0.0001; one-sample Student’s *t* test) (**b**). Effects of i.c.v. nesfatin-1 administration on sucrose preference assessed in a short-lasting 2-bottle choice procedure coupled to optogenetic stimulation of VTA dopamine neurons in ChR2^+^ mice (*N* = 8) under different metabolic conditions. The laser was kept ON and mice received 1-sec-long pulse every 3 licks at the sucralose bottle. Effects of 6–8 h fasting and i.c.v. administration of nesfatin-1 on sucrose preference (**c**), number of licks at each bottle (**d**), total number of licks (**e**) and infrared beam breaks (**f**). Behavioral sessions were performed in the second half of the dark phase, began 30 min after drug administration and lasted 20 min. Data represent mean ± SEM. ^#^*P* < 0.1, *P < 0.05, ***P < 0.001 and ****P < 0.0001 (Tukey’s *post hoc* test).

ChR2^+^ mice were then moved back onto the 2-bottle choice procedure and the effects of sucralose and laser stimulation on sucrose preference were assessed by progressively changing the configuration over 4 days (Fig. 5a). Significant effects of sucralose (*F*[1,7] = 7.61, *P* < 0.05), laser stimulation (*F*[1,7] = 51.11, *P* < 0.001) and interaction sucralose × laser stimulation were observed (*F*[1,7] = 32.47, *P* < 0.001) (Fig. 5b). Optogenetic stimulation of VTA dopamine neurons resulted in a significant drop of the preference for sucrose when using the configuration sucrose against water + laser ON (from 84.6% on day 1 to 53.3% on day 2) and sucrose against sucralose + laser ON (from 81.7% from day 3 to 19.9% on day 4) (Figs. 5b and S5). Thus, only the combination of sucralose and laser stimulation was capable to completely revert the animals´ preference for sucrose.

### I.c.v. nesfatin-1 reduces the reward value of sucrose

Finally, we then used this short-lasting 2-bottle choice procedure to address the effect of nesfatin-1 on the reward value of sucrose, using the configuration sucrose against sucralose + laser ON, in which the reward value of sucrose was opposed to that of the reference stimulus “sucralose + laser ON” under *ad libitum* and fasting conditions. I.c.v. nesfatin-1 significantly affected the preference for sucrose (*F*[2,14] = 41.00, *P* < 0.0001; Fig. 5c). Fasted PBS-treated mice displayed a significant increase in the preference for sucrose with respect to *ad libitum* fed animals (from 19.0% to 50.8%; *P* < 0.0001). Strikingly, when fasted mice were treated with i.c.v. nesfatin-1, preference for sucrose decreased significantly with respect to fasted PBS-treated animals (from 50.8% to 31.7%; *P* < 0.001).

I.c.v. treatment significantly affected both the number of licks at the sucralose + laser ON bottle (*F*[2,14] = 6.25, *P* < 0.05) and at that of sucrose (*F*[2,14] = 14.94, *P* < 0.001; Fig. 5d). Fasted PBS-treated mice showed a significant increase in the number of licks at both sucralose + laser ON (+93%; *P* < 0.05) and sucrose bottle (+725%, *P* < 0.001) as compared with *ad libitum* fed condition. Importantly, upon i.c.v. administration of nesfatin-1 in fasted mice, only the number of licks at the sucrose bottle was significantly reduced (−47.2%, *P* < 0.05), whereas those to the sucralose + laser ON bottle remained unaltered (−4.5%, *P* = 0.952), indicating once again the capability of nesfatin-1 to suppress sucrose intake in a selective manner. These data now suggest that nesfatin-1 plays a role in determining the reward value of food.

Additional results indicate that the effect of nesfatin-1 on the preference for sucrose were specific. I.c.v. treatment significantly affected the total number of licks (*F*[2,14] = 12.24, *P* < 0.001; Fig. 5e). Fasted PBS- and nesfatin-1-treated mice showed a prominent increase in the total number of licks as compared with *ad libitum* fed animals (+220% and +135%, respectively). Importantly, the number of total licks of fasted mice that were treated with nesfatin-1 did not differ with respect to PBS-treated mice in the same metabolic condition. Finally, i.c.v. drug administration did not alter the number of beam breaks (*F*[2,14] = 0.30, n.s.; Fig. 5f).

## Discussion

The gastric- and adipose tissue-derived peptide NUCB2/nesfatin-1 was previously shown to potently suppress chow food intake in rodents. Most of the studies aiming at clarifying the mechanisms underlying the anorexigenic effects of nesfatin-1 mainly focused on homeostatic centers (e.g., hypothalamus and brainstem) where this peptide is highly expressed. However, whether nesfatin-1 affects energy intake by modulating neural circuits outside of these homeostatic centers and, subsequently, behavioral aspects of food intake is largely unknown. The main findings of the present study show that NUCB2/nesfatin-1 is expressed in both dopamine and GABA neurons of the VTA and that ex vivo nesfatin-1 inhibits dopamine, but not GABA, neurons of this area, likely through the opening of potassium channels. Additionally, central nesfatin-1 reduced motivation for sucrose as assessed through the progressive ratio schedule, as well as reward sensitivity as assessed through a 2-bottle choice procedure. Furthermore, in a 2-bottle choice procedure coupled to optogenetic stimulation of VTA dopamine neurons, i.c.v. nesfatin-1 abolished the fasting-induced increase in the reward value of sucrose. While we identified the VTA as one of the regions targeted by nesfatin-1 for the regulation of food-motivated behavior, other brain areas (e.g., striatum) may also be engaged in inducing nesfatin-1´s effects on the reward value of food. Collectively, these findings provide evidence for a thus far unidentified role of NUCB2/nesfatin-1 system in the modulation of the brain reward system and hedonic aspects of food intake.

### NUCB2/nesfatin-1 is expressed in the VTA and hyperpolarizes dopamine, but not GABA, neurons

NUCB2/nesfatin-1 expression was previously described in some reward-related brain areas [13]; to the best of our knowledge, we now demonstrate for the first time that NUCB2/nesfatin-1 is expressed in the VTA by both dopamine and GABA neurons, and suggest that dopamine neurons may be the primary source of nesfatin-1 in this area. A limitation of the present study, however, is the use of calretinin as a marker for GABA neurons. In fact, this calcium-binding protein is expressed not only by GABA but also by dopamine [39, 41] and, to a lesser extent, by glutamate neurons [42], thus potentially impairing the quantification of calretinin-NUCB2/nesfatin-1 co-expression.

Additionally, we show that nesfatin-1 inhibited putative dopamine, but not GABA, neurons of the VTA most likely through the activation of potassium channels as the nesfatin-1-induced outward current was fully blocked by the application of barium chloride, a specific blocker of these channels [43, 44]. Moreover, in Li et al. [26] and in the present study, nesfatin-1 was observed to exert inhibitory effects while both glutamate and GABA_A_ receptor signaling was blocked, pointing towards a direct action of nesfatin-1 on dopamine neurons independently of excitatory or inhibitory inputs. However, since GABA_B_ receptor signaling was not blocked, it remains to be investigated whether nesfatin-1 induces potassium currents through the modulation of GABA release and GABA_B_ receptors signaling. Instead, we observed no effects in the activity of VTA GABA neurons upon nesfatin-1 application, suggesting that the necessary machinery to respond to it may not be expressed by these neurons. Our data indicate that nesfatin-1 negatively modulates the activity of VTA dopamine neurons and we speculate that nesfatin-1, released by both dopamine and GABA neurons, may act in an autocrine/paracrine manner on dopamine neurons. The understanding of the exact circumstances under which nesfatin-1 is released by these two neuronal populations lies outside the scope of the present study and remains to be investigated.

### Nesfatin-1 reduces motivation and preference for sucrose

We additionally show that i.c.v. nesfatin-1 dose-dependently reduces the motivation for sucrose, and that it does so without affecting locomotor behavior. While in behavioral neuroscience food restriction/deprivation regimens are commonly exploited to enhance the appetite and motivational state of animals engaged in an operant task, we specifically evaluated operant responding for food reward in *ad libitum* fed animals to better separate motivational aspects from general appetite. Additionally, similarly to a previous report [34], we found that chow food intake was not affected at the time point at which motivation for food reward was reduced (i.e., 0–1.5 h; Fig. 2d). The latter observation suggests that nesfatin-1 could act in areas other than brain sites primarily involved in homeostatic appetite regulation, e.g., hypothalamus and brainstem. Given the key role of the VTA in reward processing and motivated behavior, and based on our histological and electrophysiological findings, we then further corroborated this data through VTA-specific injections and, strikingly, found that nesfatin-1 acts within this area to decrease motivation for sucrose, without affecting locomotor behavior and chow food intake. As both VTA→NAcc dopamine projections and NAcc-specific D_2_ receptors activation enhances motivation for sucrose [45, 46], the effects of nesfatin-1 on food motivation are likely to result from a downregulated dopamine signaling in the NAcc. This hypothesis is in agreement with previous findings demonstrating that i.c.v. nesfatin-1 reduces dopamine release in the NAcc [25]. Noteworthy, when mice were tested under the fixed ratio 1 schedule of reinforcement, we found no effects following both i.c.v. and VTA-specific nesfatin-1 administration, suggesting that it reduces motivation for food rewards only in high-effort condition.

Our results showing the lack of significant reduction in chow food intake following VTA-specific administration of nesfatin-1 at first glance might appear to contrast with those of a previous study, where chow food intake was reduced already 1 h after administration of nesfatin-1 into the VTA [25]. In the latter study, however, nesfatin-1 was administered bilaterally, whereas in the present study mice were injected unilaterally. Overall, our findings clearly demonstrate that nesfatin-1 decreases energy intake by reducing the animals´ motivation to acquire food, an effect independent of homeostatic food intake regulation.

Employing a long-lasting 2-bottle choice procedure to assess sucrose preference in the home cage, we found that i.c.v. nesfatin-1 reduces sucrose intake and preference, suggesting a role for nesfatin-1 in modulating the sensitivity to rewarding food. Similar results were recently obtained by another research group following i.c.v. administration of nesfatin-1 midfragment in rats [47]. Even though i.c.v. nesfatin-1 was shown to reduce also water intake [48], in the present study water intake was not affected by the treatment, suggesting a specific effect of nesfatin-1 on sucrose intake rather than a general suppression of ingestive behaviors.

While in this study we observed a reduction in motivated behavior, sucrose intake and preference, in the study by Merali and colleagues i.c.v. administration of nesfatin-1 did not affect palatable snacks intake when rats were placed in a familiar environment [49]. This apparent difference between ours and Merali´s findings could be attributed to differences in the experimental procedures (effort- and choice-based vs. single choice), duration (1 h and 4 h vs. 15 min) and type of reinforcer employed (liquid sucrose vs. commercial palatable food).

### Nesfatin-1 decreases the reward value of sucrose

To elucidate whether the reduction in sucrose intake and preference observed in the long-lasting 2-bottle choice was due to a decrease in general appetite, taste palatability and/or reward value of sucrose, further experiments were performed. To account for general appetite, we coupled optogenetics with a short-lasting 2-bottle choice procedure to assess sucrose preference at a time point at which appetite was unaffected. To circumvent the impact of taste and specifically assess the reward value of sucrose under different metabolic conditions, we made use of a reference stimulus as one possible choice which consisted of a non-caloric sucralose solution providing taste, the ingestion of which was contingent to laser stimulation of VTA dopamine neurons, in turn leading to a tonic dopamine release [27]. The second possible choice consisted of a bottle containing a sucrose solution, the ingestion of which is known to induce dopamine release [50], and whose reward value was compared to that of the reference stimulus. Here we show that mice fed *ad libitum* highly preferred the sucralose solution coupled with optogenetic stimulation of VTA dopamine neurons. Fasted mice, with presumably low levels of endogenous NUCB2/nesfatin-1 [2, 4, 16, 51, 52], showed a significant increase in the sucrose preference, indicating an enhancement in the reward value of sucrose. Strikingly, the latter effect was fully abolished by i.c.v. administration of nesfatin-1, with animals showing a reduced sucrose preference due to a selective suppression of sucrose intake, indeed suggesting that nesfatin-1 decreases the reward value of sucrose.

This observation is to be attributed to a reduction of the post-ingestive effects of sucrose induced by nesfatin-1 rather than an action at the level of the taste receptors. In fact, despite in the current study sucralose was more palatable than sucrose, mice highly preferred sucrose over sucralose (81.7%; Fig. 5b), likely due to its energy content. This is in agreement with previous findings showing the development of conditioned behavior and enhanced dopamine release in the NAcc of taste receptor knock-out mice upon sucrose intake, but not sucralose, indeed suggesting that animals can sense the caloric value of nutrients independently of their taste [50]. As a consequence of higher sucrose intake, fasted PBS-treated mice displayed a strong increase in blood glucose levels (116.5 ± 16.0 vs. 176.4 ± 17.8 mg/dl; *t*[7] = 13.36, *P* < 0.00001), which is likely to increase dopamine release as was previously observed [50, 53, 54]. It can be assumed that nesfatin-1 reduces the activation of midbrain dopamine neurons and prevents the sucrose-induced release of dopamine, therefore resulting in anti-rewarding effects. Importantly, we did not observe a reduction in the number of total licks and beam breaks, suggesting that nesfatin-1 neither induces taste aversion and/or malaise, nor reduces appetite at this specific time interval (30-50 min after drug administration).

It can be argued that the optogenetic 2-bottle choice procedure is not optimal for pinpointing the brain areas mediating nesfatin-1´s effects. In fact, since nesfatin-1 was administered i.c.v., the involvement of other brain areas cannot be excluded. Moreover, the employment of optogenetic stimulations as a reference stimulus will alter the normal functions of the VTA dopamine neurons and thus possibly masking nesfatin-1´s effects.

### Working model and conclusions

Even though our ex vivo electrophysiology experiments did not specifically test causal mechanisms underlying the observed behavioral changes, the current study suggest that nesfatin-1 influences energy intake *via* the modulation of the mesolimbic dopamine system. As a working model, we propose that NUCB2/nesfatin-1, of peripheral and/or of central origin, exerts its anti-rewarding effects by interacting with a putative Gαi/o protein-coupled receptor [10] to hyperpolarize dopamine neurons.

As a working model, we propose that NUCB2/nesfatin-1 can be released by peripheral organs, such as the stomach, and reach the CNS via the bloodstream. Alternatively, NUCB2/nesfatin-1 can be released by neurons of the VTA or other brain areas and interact with a putative Gαi/o protein-coupled receptor10 to hyperpolarize neighboring dopamine neurons. Thus, NUCB2/nesfatin-1 participates in assigning the value of a reinforcer and the willingness to exert effort to obtain it.

Obesity represents one of the most significant world health problems as it has reached pandemic proportions with one of the major drivers clearly being protracted excess calorie intake [55]. In turn, habitual overeating might be triggered by obesogenic factors, such as the ready availability of fat- and sugar-rich foods [24]. Thus, the observation that nesfatin-1 decreases behavioral aspects of food intake by acting within the midbrain dopamine system is of great interest as nesfatin-1 injected centrally or peripherally was shown to decrease food intake in animal models of obesity [56–58], indicating that the response to nesfatin-1 is preserved even in the state of leptin resistance. In addition, nesfatin-1 not only promotes a negative energy balance by reducing energy intake and increasing energy expenditure, but also decreases gastrointestinal functions and improves insulin sensitivity [1]. These pleiotropic actions together with the present findings render the NUCB2/nesfatin-1 system an appealing target for the development of novel therapeutical treatments towards obesity.

## Funding and disclosure

Open access funding provided by Projekt DEAL. The authors declare no competing financial interests.

## Supplementary information

Supplementary Material
