# Supplementary material for: Nesfatin-1 decreases the motivational and rewarding value of food
Source: Neuropsychopharmacology. 2020 Apr 30;45(10):1645–55. doi: 10.1038/s41386-020-0682-3 (PMC7419560; doi:10.1038/s41386-020-0682-3)
Supplement: Supplementary file 1 — Supplementary Material [file 41386_2020_682_MOESM1_ESM.docx]

**Supplementary Material**

**Nesfatin-1 Decreases the Motivational and Rewarding Value of Food**

Riccardo Dore, Ph.D.^1,2,*^, Regina Krotenko^1,2,#^, Jan Philipp Reising^1,2,#,§^, Luca Murru, Ph.D.^3^, Sivaraj Mohana Sundaram, Ph.D.^2,4^, Alessandro Di Spiezio, Ph.D.^2,4,&^, Helge Müller-Fielitz, Ph.D.^2,4^, Markus Schwaninger, M.D.^2,4^, Olaf Jöhren, Ph.D.^2^, Jens Mittag, Ph.D.^1,2^, Maria Passafaro, Ph.D.^3^, Marya Shanabrough^5^, Tamas L. Horvath^5,6^, Carla Schulz, Ph.D.^1,2,$^, Hendrik Lehnert, M.D.^1,2,$^

^1^Department of Internal Medicine I, University of Lübeck, Ratzeburger Allee 160, 23562 Lübeck, Germany; ^2^Center of Brain, Behavior and Metabolism (CBBM), University of Lübeck, Ratzeburger Allee 160, 23562 Lübeck, Germany; ^3^CNR, Institute of Neuroscience, 20129, Milan, Italy; ^4^Institute for Experimental and Clinical Pharmacology and Toxicology, University of Lübeck, Ratzeburger Allee 160, 23562 Lübeck, Germany; ^5^Department of Comparative Medicine, Program on Integrative Cell Signaling and Neurobiology of Metabolism, Yale University School of Medicine, New Haven, CT 06520, USA; ^6^Department of Anatomy and Histology, University of Veterinary Medicine, Budapest, H-1078, Hungary.

Present addresses: ^§^Department of Women´s and Children´s Health, Karolinska Institutet, Stockholm, 171 76, Sweden; ^&^Department of Biochemistry, University of Kiel, Kiel, 24118, Germany.

^#^These authors equally contributed to this work

^$^These authors share senior authorship

Correspondence and requests for materials should be addressed to:

Riccardo Dore ([riccardo.dore@uksh.de](mailto:riccardo.dore@uksh.de))

Department of Internal Medicine I

Center of Brain, Behavior and Metabolism (CBBM)

University of Lübeck

Ratzeburger Allee 160

23562 Lübeck, Germany

Phone: +49 451 3101 7821

**Supplementary Materials and Methods**

**Double-fluorescence Immunohistochemistry**

Wild type *ad libitum* fed mice were deeply anesthetized (pentobarbital 320 mg/kg, i.p.) andtranscardially perfused with Ringer’s solution and then with 4%. Coronal sections (40 μm) were cut at the vibratome (Leica, Germany), washed three times in PBS and permeabilized with 0.2% Triton X-100 in PBS (PBS-TX) for 20 min. VTA-containing free-floating sections were then incubated with 3% normal goat serum in PBS-TX for 1 h and subsequently incubated with rabbit anti-NUCB2/nesfatin-1 polyclonal antibody (Phoenix Pharmaceuticals, US; #H-003-22, 1:1000), mouse anti-TH monoclonal antibody (Sigma Aldrich, US; #T1299, 1:500), goat anti-calretinin polyclonal antibody (Millipore, US; #AB1550, 1:1000) and mouse anti-glutamate decarboxylase 67 (GAD67) (Millipore, US; #MAB5406, 1:1000) for 12 h at 4°C with gentle shaking. After washing three times with PBS for 10 min, for the NUCB2/nesfatin-1 – TH co-staining sections were incubated with goat anti-rabbit Alexa 633 (Invitrogen, US; #A-21071,1:500) and goat anti-mouse Alexa 488 (Invitrogen, US; #A-31619, 1:500) antibodies as well as DAPI (1 μg/ml) for 2 h at room temperature with gentle shaking. For the NUCB2/nesfatin-1 – calretinin co-staining, sections were incubated with donkey anti-rabbit Alexa 647 (Invitrogen, US; #A-31573, 1:500) and with donkey anti-goat Alexa 488 (Invitrogen, US; #A-11055) antibodies as well as DAPI (1 μg/ml) for 2 h at room temperature with gentle shaking. For the NUCB2/nesfatin-1 – GAD67 co-staining, sections were incubated with donkey anti-rabbit Alexa 647 (Invitrogen, US; #A-31573, 1:500) and with donkey anti-mouse Alexa 488 (Invitrogen, US; #A-21202, 1:500) antibodies as well as DAPI (1 μg/ml) for 2 h at room temperature with gentle shaking. Next, sections were washed three times with PBS for 10 min and mounted on glass slides and covered with Mowiol 4-88 (Carl Roth, Germany). After acquisition of fluorescence images on a Leica SP5 confocal microscope, with regards to NUCB2/nesfatin-1 – TH and NUCB2/nesfatin-1 – calretinin co-stainings, we quantified and summed up the number of neurons from two non-consecutive sections at approximately -3.40 mm from the bregma and % of colocalization was calculated for each mouse. The mean percent of colocalization of 5 mice was finally calculated. Neurons counting was confirmed by two independent examiners and performed with Image J software (NIH).

***Ex vivo* Electrophysiology**

Horizontal brain slices containing the VTA were prepared from C57BL/6J mice brain following standard procedures with minor modifications^1^. Mice were sacrificed by cervical dislocation, brains were dissected and placed in an ice-cold cutting solution containing (in mM) 220 sucrose, 2 KCl, 1.3 NaH_2_PO_4_, 12 MgSO_4_, 0.2 CaCl_2_, 10 glucose, 2.6 NaHCO_3_ and 3 kynurenic acid (pH 7.3, equilibrated with 95% O_2_ and 5% CO_2_). Horizontal brain slices (250 μm) containing the VTA were cut at the vibratome (VT1000S; Leica, Germany) and right after incubated at 33°C in standard aCSF solution containing (in mM): 125 NaCl, 2.5 KCl, 1.25 NaH_2_PO_4_, 1 MgCl_2_, 2 CaCl_2_, 25 glucose and 26 NaHCO_3_ before recordings. Slices were then transferred to a recording chamber and constantly superfused with aCSF at a rate of ~2 ml/min at 33°C. Whole-cell patch clamp recordings were performed under an infrared-differential interference contrast microscope (Nikon Instruments Europe BV, The Netherlands) and using a Multiclamp 700B amplifier (Molecular Devices, US). Patch borosilicate glass capillaries with filament (1.5 μm, 3-5 3–5 MΩ; Sutter Instruments, US) were prepared with a 4-step horizontal puller (P-1000; Sutter Instruments, US) and filled with internal solution containing (in mM) 126 K gluconate, 4 NaCl, 1 EGTA, 1 MgSO_4_, 0.5 CaCl_2_, 3 ATP (Mg salt), 0.1 GTP (Na salt), 10 glucose, 10 HEPES–KOH (pH 7.3, equilibrated with 95% O_2_ and 5% CO_2_). Putative dopamine and GABA neurons were identified electrophysiologically by sag potential amplitude as previously described^2^ but with 20 pA steps to elicit I*_h_*-related sag potentials and evoke action potential firing. VTA putative dopamine and GABA neurons were then clamped at a holding potential of −50 mV and slices were superfused with aCSF supplemented with kynurenic acid (3 mM) and bicuculline (20 μM) to block glutamatergic transmission and GABA_A_ receptors signaling, respectively. After a ~3-min baseline period recording, slices were acutely treated with human recombinant nesfatin-1 (10 nM; dissolved in aCSF) and the *I* holding current shift was recorded. Dose of nesfatin-1 was based on previous studies^3,4^. At the end of each recording, slices were superfused with the potassium channels inhibitor BaCl_2_ (1 mM) to confirm the specificity of nesfatin-1-induced current. Experiments were analyzed offline with Axon Clampfit 10.1 software (Molecular Devices, US).

**Intracranial Surgery and Microinjection Procedures**

The surgical procedure was performed as previously described for rats^5^. Under anesthesia (ketamine 80 mg/kg and xylazine 12 mg/kg, i.p.), mice underwent unilateral implantation of a 26-gauge stainless steel cannula (PlasticsOne, US) under stereotaxic control (Kopf Instruments, US). The following coordinates were used (relative to bregma, in mm): lateral ventricle, AP: -0.22, ML: ±1.00, DV: -1.50 from the skull surface; VTA, AP: -3.40, ML: 0.35, DV: -3.80 from the skull surface. Incisor bar was set at -2.00 mm below the interaural line, according to Paxinos and Franklin^6^. Two stainless steel screws were fastened to the mice´ skull around the cannula, and denture acrylic (Paladur®; Heraeus, Germany) was applied forming a pedestal that firmly anchored the cannula. Mice were allowed to recover for a minimum of 5 days and injected with carprofen (5 mg/kg, s.c., twice daily) on the day of the surgery as well as for the three following days. On the experimental day, the dummy cannula was removed to insert an injector (0.80 mm protrusion for i.c.v. and 0.40 mm protrusion for VTA) into the guide cannula. Injection of substances (1 µl for i.c.v. and 0.5 µl for VTA) was performed in freely moving animals by a 5 µl Hamilton syringe (Hamilton, Switzerland) and infusion pump over 2 minutes. The injector was left inserted for an additional minute to prevent backflow. Upon recovery from surgery, i.c.v. cannula placement was confirmed by a positive dipsogenic response to angiotensin II (15 pmol/µl; Sigma Aldrich, US). All the animals showed a water intake of ~600 µl (~300 licks) 15 minutes after injection. VTA cannula placement was confirmed at the end of study by a site-specific injection of Evans blue 1% (0.5 µl) and one animal was excluded from final analysis study due to cannula misplacement.

**Fixed Ratio and Progressive Ratio Schedule of Reinforcement for Sucrose**

The following procedure was similar to that previously described for rats^7^. Mice first underwent an overnight session (16-18h) under a fixed ratio 1 schedule of reinforcement in two-lever operant conditioning chambers (MedAssociates, US), during which food and water were not provided. One press at the active lever led to the delivery of one reward (20 μl of sucrose solution 584 mM; ~20%) into a receptacle, whereas presses at the inactive lever had no programmed consequences. From the following day on and at the onset of the dark phase, mice underwent a 60-min daily session under a fixed ratio 1 schedule of reinforcement for 5 days. Next, mice were moved onto a fixed ratio 3 schedule of reinforcement, in which 3 presses at the active lever were required to obtain one reward. After 5 days, a progressive ratio schedule of reinforcement was introduced, in which the number of active lever presses required to obtain successive rewards increased within the session according to the following shallow exponential equation: response ratio=[4·(e^# of reinforcer×0.075^)-3.8], rounded to the nearest integer. Sessions ended either after 60 min or prematurely if mice had not pressed the active lever for at least 10 min, whichever came first. Immediately after the end of the session, mice were removed from the chambers and returned to their home cage with food and water freely available. Number of active lever presses, breakpoint (the maximum number of presses emitted by a mouse to obtain the last reward), number of rewards earned during the session and session duration (the time between mice were placed into the chambers and the end of the session) were recorded as measures of motivation. Number of inactive lever presses and inactive lever presses rate [number of inactive lever presses / session duration (min) × 10 (min)] were also recorded as measures of locomotion and/or exploratory behavior. Experimental testing began upon reaching stable performance (<15% variation in the breakpoint across 3 consecutive days). On testing day, to minimize any handling-related impact on the animals´ performance, mice received a central administration of nesfatin-1 (i.c.v.: 100 and 300 pmol; VTA-specific: 50 pmol) or PBS 30 min prior to behavioral sessions. In a control experiment, to ensure that nesfatin-1 effects were specific for high-effort condition, a new set of mice received a central administration of nesfatin-1 (i.c.v.: 300 pmol; VTA-specific: 50 pmol) and undergo the fixed ratio 1 schedule of reinforcement. The experiments were performed in a counterbalanced design and with at least 3 days of washout period.

**Virus Injection and Fiber Optic Implantation**

To achieve dopamine neuron-specific expression of the photosensitive ion channel Channelrhodopsin-2 (ChR2), DAT-Cre mice were anesthetized (ketamine 80 mg/kg and xylazin 12 mg/kg, i.p.) and stereotaxically injected unilaterally with 1 µl of purified Cre recombinase-dependent viral vector rAAV-FLEX-*rev*-ChR2-TdTomato (ChR2^+^) or rAAV-FLEX-*rev*-TdTomato vector (ChR2^-^)^8^ (titer: 5.11 × 10^9^ genomic particles/µl) in the VTA (relative to bregma, in mm: AP -3.40; ML -0.35; DV -4.20 from the skull surface) through a pulled-glass micropipette over ~2 minutes. The micropipette was left in place for additional 8 minutes to minimize backflow. A fiber optic (200-μm core, 0.39 NA; Thorlabs, US) was then implanted above the VTA (DV -4.00 from the skull surface). Finally, a 26-gauge guide cannula was implanted aiming at the lateral ventricle as described above. Fiber optic and guide cannula were then anchored to two stainless steel screws fastened to the mice´ skull with the application of denture acrylic (Figure 4A). Mice were allowed to recover for a minimum of 5 days and injected with carprofen (5 mg/kg, s.c., twice daily) on the day of the surgery as well as for the three following days.

**Immunofluorescence in DAT-Cre-ZsGreen Mice**

Three weeks after the virus injection into the VTA, DAT-Cre-ZsGreen mice were deeply anesthetized (pentobarbital 320 mg/kg, i.p.) and transcardially perfused with Ringer’s solution and then with 4% PFA. Brains were dissected and postfixed in 4% PFA at 4°C overnight. 50-µm sections were cut at the vibratome (Leica, Germany) and kept in PBS and 0.02% sodium azide at 4°C. Free-floating sections were washed twice in Tris-buffered saline (TBS) and incubated for 15 minutes with DAPI (1 µg/ml TBS). After two additional washing steps in TBS for 5 minutes, sections were mounted on glass slides, covered with Mowiol 4-88 and acquired with a fluorescent microscope (Leica, Germany).

**Optogenetic Setup**

The optogenetic setup was similar to that previously described by Domingos and colleagues^9-11^. At least 3 weeks after surgery (to allow for sufficient viral infection), DAT-Cre mice were tethered to a rotary joint patch cable through a mating sleeve (Thorlabs, US). The other terminal end of the rotary joint patch cable was connected to a shutter (Doris Lenses Inc, Canada) that was in turn controlled by a Master-8 pulse generator (A.M.P.I., Israel). The shutter was connected to a 473-nm blue DPSS laser (100mW; Shanghai Laser & Optics Century Co., Ltd., China; output power: 25-30 mW). Finally, lickometers of MedAssociates behavioral chambers were controlled by MedPC *via* a transistor-transistor logic impulse, and were connected to the Master-8 pulse generator. Photostimulation was delivered for 1 second every 3 licks at the designated “laser bottle”. Such an optogenetic stimulation pattern (1-second stimulation) was previously used in similar behavioral tasks in mice^9-12^ and was shown to produce a stronger excitatory effect than stimulations at 1, 5, 10 and 20 Hz^12^. Finally, behavioral chambers were also equipped with three horizontal infrared photo beams to monitor locomotor activity.

**Behavioral Validation of Optogenetics: 2-bottle Choice and Conditioning Procedures**

To assess the reinforcing efficacy of the optogenetic activation of VTA dopamine neurons, we employed the 2-bottle choice and conditioning procedures. A positive response to these two behavioral procedures was used as inclusion criteria for the subsequent behavioral tests.

*2-bottle choice.* ChR2^+^ and ChR2^-^ mice underwent daily 60-minute sessions for 4 consecutive days and with 2 bottles of water available simultaneously, one of which was coupled to laser stimulation (“laser bottle”) (Figure S4C). Mice received a 1-sec-long pulse every 3 licks at the laser bottle, whose position was changed every day to account for side preference. The preference for the laser bottle was calculated for each session and then averaged for the 4 testing days as follows: laser bottle licks (n) / total licks (n) × 100.

*Conditioning.* ChR2^+^ and ChR2^-^ mice underwent a conditioning procedure^13^, which consisted of training and testing phase (Figure S4F). After assessing side preference in a baseline session, during the training days mice were conditioned to the less preferred side of the chamber for 3 days/side by undergoing one-bottle forced choice 60-min sessions. For instance, on day 1, mice had free access to water + laser ON (on one side of the chamber). On day 2, mice had free access to water + laser OFF (on the opposite side of the chamber). Likewise, mice that had access to water + laser OFF on day 1 were presented with water + laser ON on day 2. Days 3 and 5 were the same as day 1, and day 4 and 6 were the same as day 2. On day 7, mice underwent a 60-min testing session with both bottles of water being present simultaneously in the chamber (laser OFF). Preference for the previous water + laser ON side was then calculated as follows: water + laser ON licks / total licks × 100. During conditioning and testing days, mice were always tethered to the rotary joint patch cable to prevent reward expectation.

**Statistical Analysis**

All statistical analyses were performed in GraphPad Prism 7.02 (GraphPad, US). Data from *ex vivo* electrophysiology was analyzed using one-way repeated measures analysis of variance (RM-ANOVA), followed by Tukey’s *post hoc* test, or using unpaired Student´s *t*-test. The effects of nesfatin-1 on progressive ratio and fixed ratio 1 schedule of reinforcement were analyzed using two-way RM-ANOVA with treatment and lever as within-subject factors; subsequently, one-way RM-ANOVA with dose as within-subject factor was performed, followed by Dunnett´s *post hoc* test for i.c.v. treatment, or using paired Student´s *t*-test. The effects of nesfatin-1 on food intake were analyzed using two-way RM-ANOVA with nesfatin-1 and time as within-subject factors; subsequently, one-way RM-ANOVA with dose as within-subject factor was performed, followed by Dunnett´s *post hoc* test. Results from the long-lasting 2-bottle choice procedure were analyzed using paired Student´s *t*-test. Data from the validation of the optogenetics and the establishment of the short-lasting 2-bottle choice procedure was analyzed using two-way RM-ANOVA with laser and sucralose as within-subject factors, followed by Tukey’s *post hoc* test, or using unpaired Student´s *t*-test. The effects of fasting and nesfatin-1 in the short-lasting 2-bottle choice procedure were analyzed using one-way RM-ANOVA, followed by Tukey’s *post hoc* test. Significance was set at *P*<0.05. All data are expressed as mean ± standard error of the mean (SEM). Experimental designs and samples sizes aimed at minimizing usage and distress of animals, and were sufficient for detecting robust effect sizes.

**Supplementary Figures and Figure Legends**

**Figure S1**


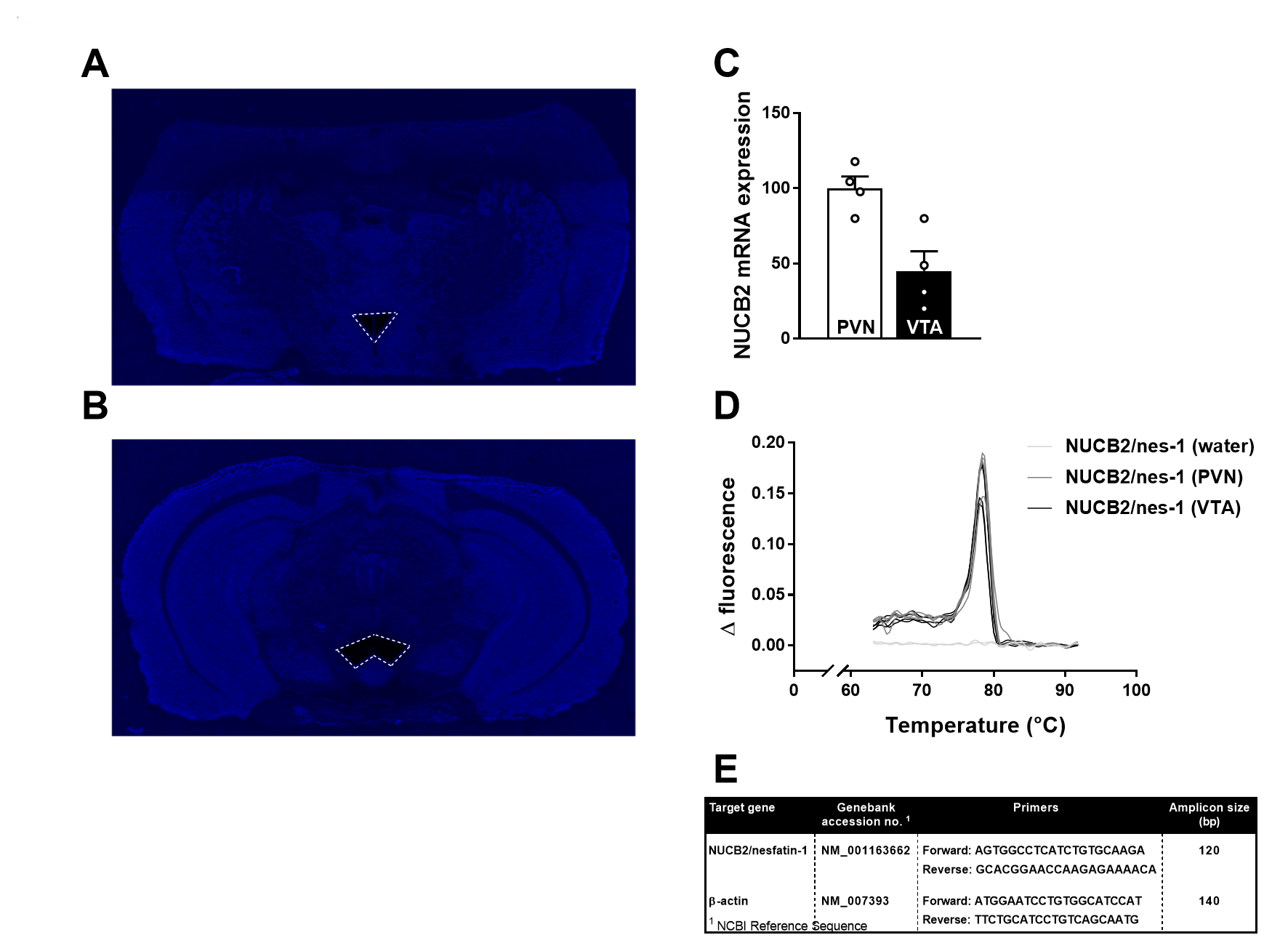


**Figure S1:**

Representative example of LCM of PVN- (A) or VTA-containing (B) brain slices from *ad libitum* fed mice (*N*=4). NUCB2/nesfatin-1 mRNA levels in the PVN and VTA (C). Specificity of qRT-PCR amplification is shown by melting (dissociation) curve analysis of β-actin and NUCB2 amplicons and non-template controls (D). Oligonucleotide primers used in qRT-PCR (E).

**Figure S2**


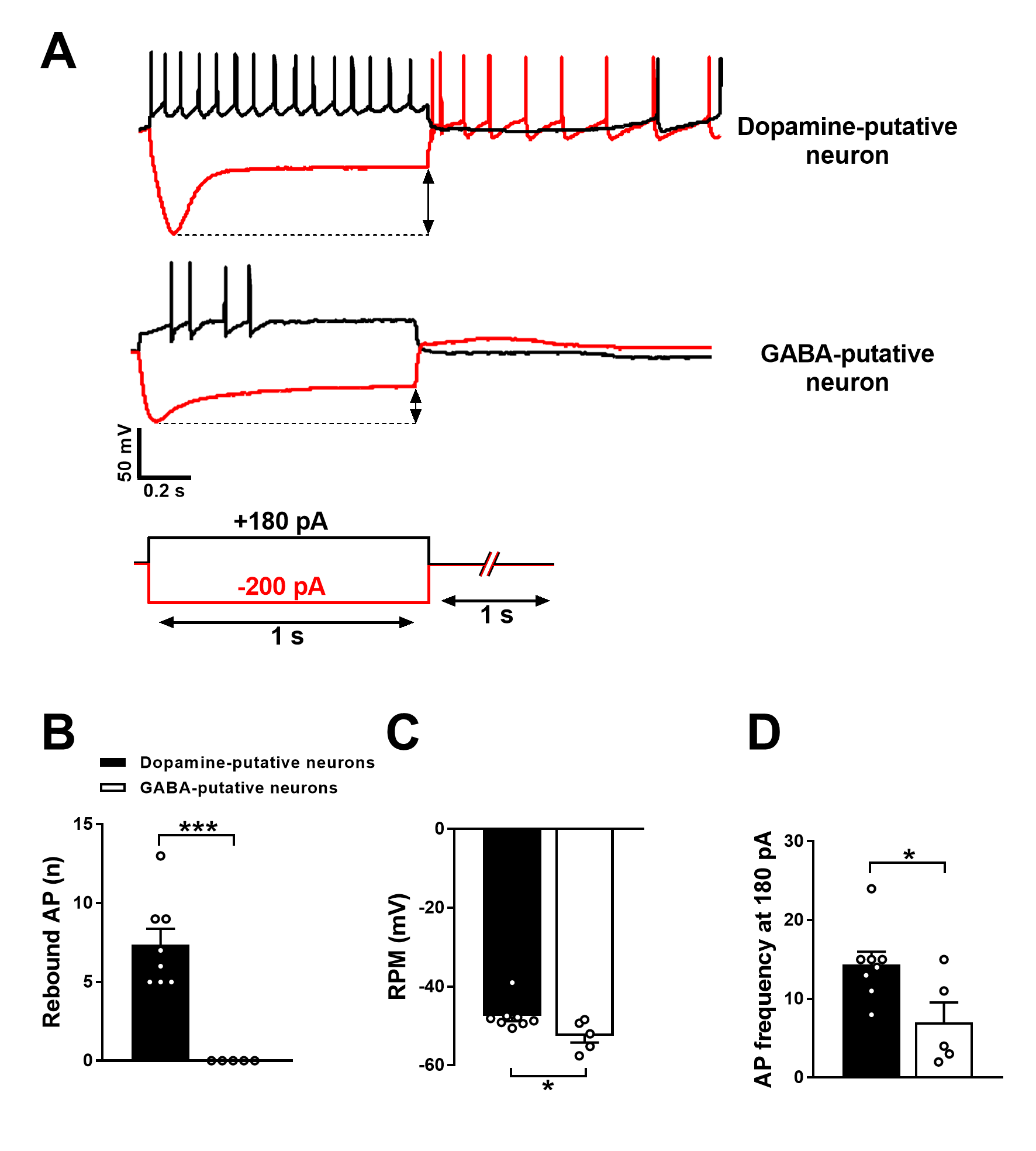


**Figure S2:**

Representative electrophysiological profiles of putative dopamine and GABA neurons of the VTA depicting the larger sag potential amplitude in dopamine neurons and the absence of rebound spikes in GABA neurons in response to hyperpolarizing current injection (-200 pA, in red; A and B), and the action potential frequency in response to depolarizing current injection (+180 pA, in black; A and D). Number of rebound action potentials in response to hyperpolarizing current injection (*t*[11]=5.74, *P*<0.0001) (B). Resting membrane potential measured before clamping the neurons at a holding potential of -50 mV (*t*[11]=2.36, *P*<0.05) (C). Action potentials frequency in response to depolarizing current injection (*t*[11]=2.57, *P*<0.05) (D) (*N*_neurons_=5-7, *N*_mice_=4). Data represent mean ± SEM. **P*<0.05 and ****P*<0.001 (Student’s *t*-test).

**Figure S3**


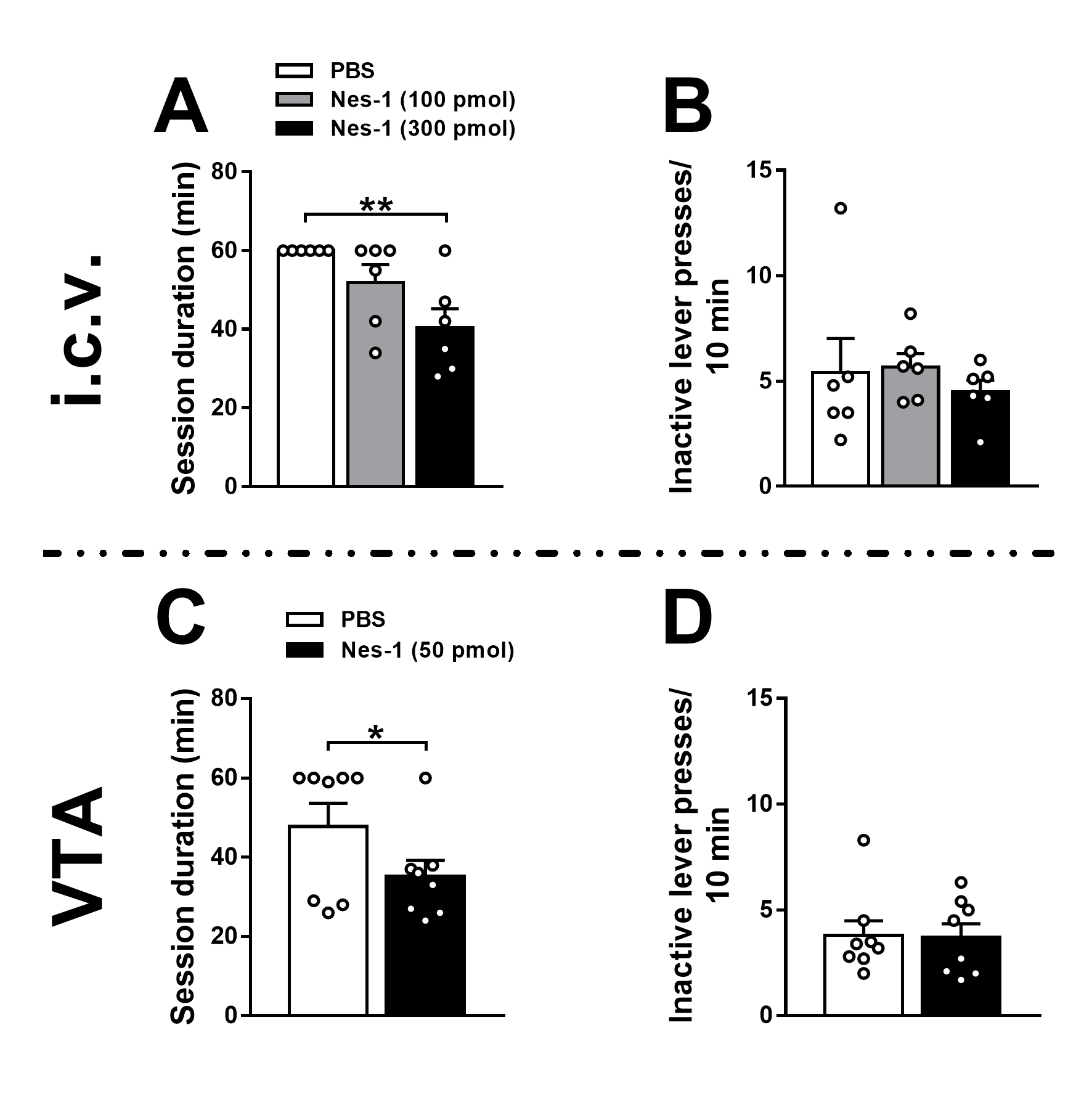


**Figure S3:**

Effects of i.c.v. administration of nesfatin-1 on the session duration (A) and inactive lever presses rate (B) in *ad libitum* fed mice in the progressive ratio schedule of reinforcement (*N*=6). Effects of VTA-specific administration of nesfatin-1 on the session duration (C) and inactive lever presses rate (D) in *ad libitum* fed mice in the progressive ratio schedule of reinforcement (*N*=8). Operant behavioral sessions were performed at the beginning of the dark phase, began 30 min after drug administration and lasted 1 h. Data represent mean ± SEM. **P*<0.05 and ***P*<0.01 *vs.* PBS group (Dunnett´s *post hoc* test or paired Student’s *t*-test).

**Figure S4**


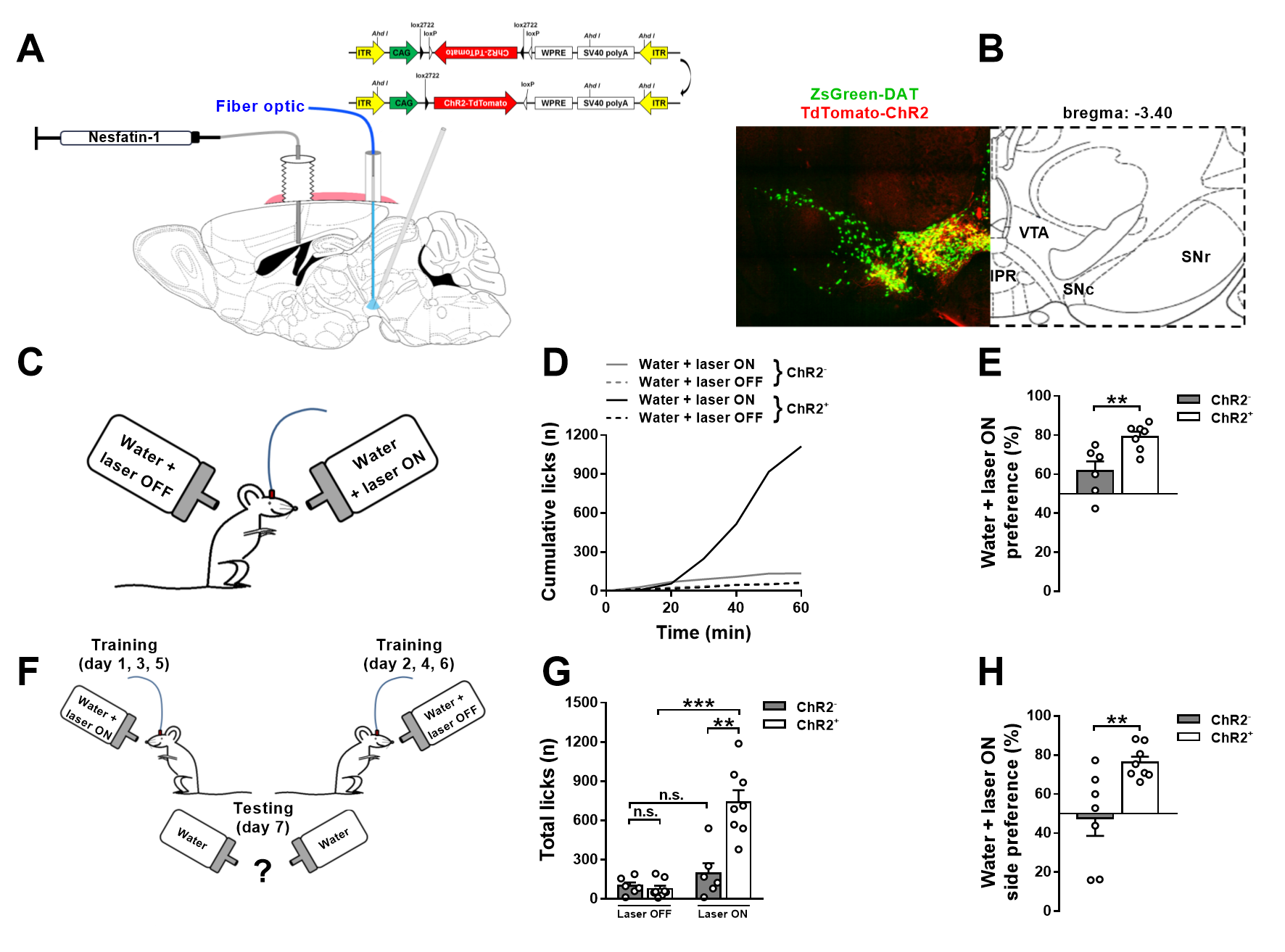


**Figure S4:**

Behavioral validation of optogenetics. Schematic representation of virus injection, fiber optic and guide cannula implantation (A). Dopamine neuron-specific ChR2 expression was achieved by infusing a Cre recombinase-dependent rAAV-FLEX-*rev*-ChR2-TdTomato vector into the VTA of DAT-Cre-ZsGreen (for *in vitro* validation) or DAT-Cre (for *in vivo* experiments) mice, which drives TdTomato-ChR2 expression in dopamine neurons (B; IPR, rostral interpeduncular nucleus; VTA, ventral tegmental area; SNc, *substantia nigra pars compacta*; SNr, *substantia nigra pars reticulata*). Schematic drawing depicting the configuration of the 2-bottle choice procedure, with ChR2^-^ and ChR2^+^ mice receiving 1-sec-long laser stimulation every three licks at the water + laser ON bottle (C). Representative 60-min behavioral session, with a ChR2^+^ mouse dramatically escalating the number of licks at the water + laser ON bottle, an effect not observed in ChR2^-^ mice (D). Mean preference (4 days) for the water + laser ON bottle (E) (*N*=6-8). Schematic drawing depicting the configuration of the conditioning procedure (F). Mean number of licks (3 days/side) at the water + laser ON and water + laser OFF bottles during the training phase (G). Mean preference for the water + laser ON side on the testing day (H) (*N*=6-8). Data represent mean ± SEM. ***P*<0.01 and ****P*<0.001 (Student’s *t*-test or Tukey *post hoc* test).

**Figure S5**


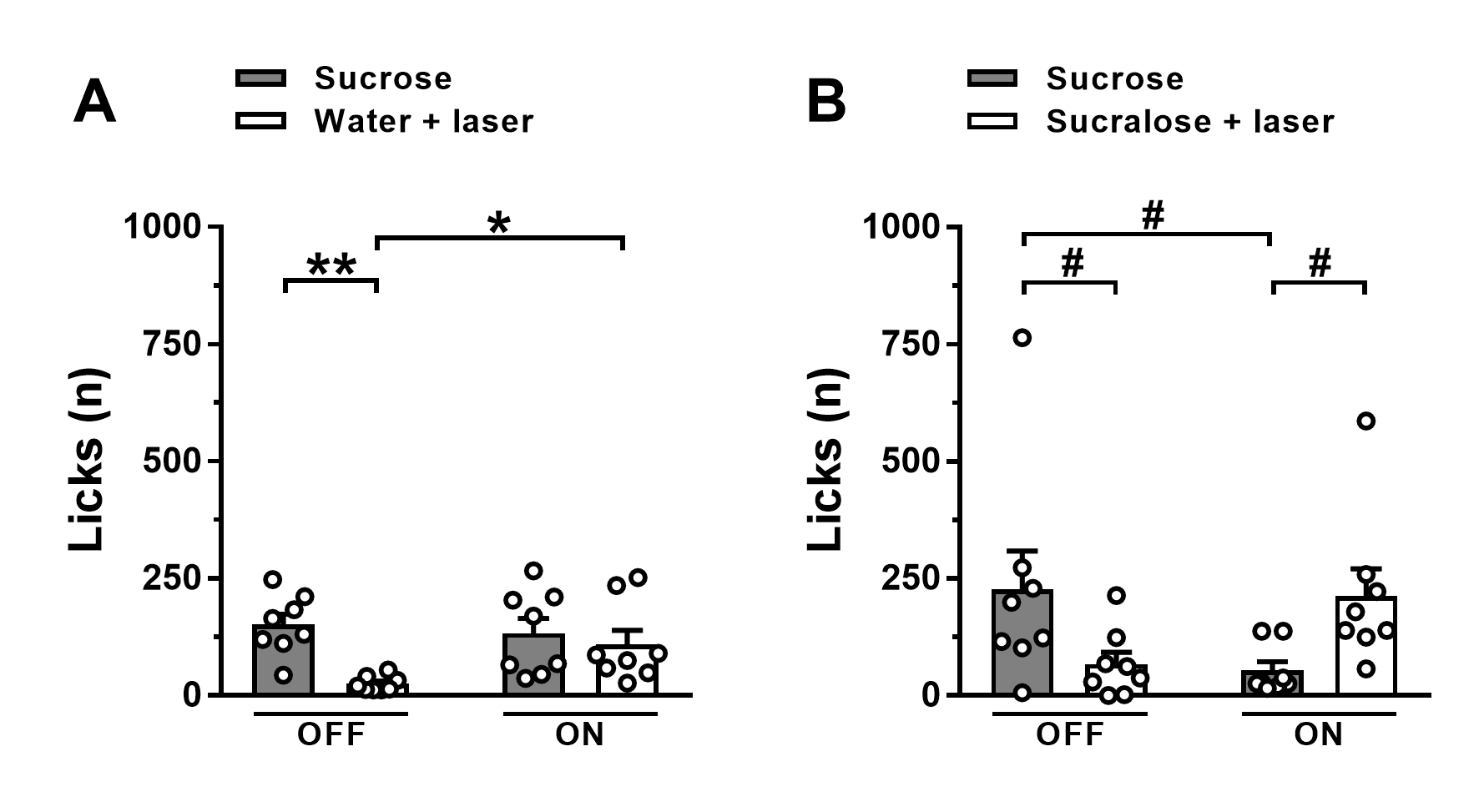


**Figure S5:**

Validation of the short-lasting 2-bottle choice procedure employing optogenetics in *ad libitum* fed mice. Number of licks relative to experiment in Figure 5A. Statistical results relative to panel A: Laser: *F*[1,7]=3.73, *P*=0.095; Solutions: *F*[1,7]=3.88, *P*=0.090; Laser × Solutions: *F*[1,7]=10.06, *P*<0.05. Statistical results relative to panel B: Laser: *F*[1,7]=0.04, n.s.; Solutions: *F*[1,7]=0.00, n.s.; Laser × Solutions: *F*[1,7]=17.71, *P*<0.01 (*N*=8). Data represent mean ± SEM. ^#^*P*<0.1, **P*<0.05 and ***P*<0.01 (Tukey *post hoc* test).

**Supplementary References**

1 Chieng, B., Azriel, Y., Mohammadi, S. & Christie, M. J. Distinct cellular properties of identified dopaminergic and GABAergic neurons in the mouse ventral tegmental area. *The Journal of physiology* **589**, 3775-3787, doi:10.1113/jphysiol.2011.210807 (2011).

2 Merrill, C. B., Friend, L. N., Newton, S. T., Hopkins, Z. H. & Edwards, J. G. Ventral tegmental area dopamine and GABA neurons: Physiological properties and expression of mRNA for endocannabinoid biosynthetic elements. *Sci Rep* **5**, 16176, doi:10.1038/srep16176 (2015).

3 Chen, X., Shu, X., Cong, Z. K., Jiang, Z. Y. & Jiang, H. Nesfatin-1 acts on the dopaminergic reward pathway to inhibit food intake. *Neuropeptides* **53**, 45-50, doi:10.1016/j.npep.2015.07.004 (2015).

4 Li, C. *et al.* Nesfatin-1 decreases excitability of dopaminergic neurons in the substantia nigra. *J Mol Neurosci* **52**, 419-424, doi:10.1007/s12031-013-0169-3 (2014).

5 Dore, R. *et al.* CRF mediates the anxiogenic and anti-rewarding, but not the anorectic effects of PACAP. *Neuropsychopharmacology* **38**, 2160-2169, doi:10.1038/npp.2013.113 (2013).

6 Paxinos, G. & Franklin, K. B. J. The Mouse Brain in Stereotaxic Coordinates. *Academic Press* (2003).

7 Cottone, P., Sabino, V., Steardo, L. & Zorrilla, E. P. Intermittent access to preferred food reduces the reinforcing efficacy of chow in rats. *Am J Physiol Regul Integr Comp Physiol* **295**, R1066-1076, doi:10.1152/ajpregu.90309.2008 (2008).

8 Atasoy, D., Aponte, Y., Su, H. H. & Sternson, S. M. A FLEX switch targets Channelrhodopsin-2 to multiple cell types for imaging and long-range circuit mapping. *J Neurosci* **28**, 7025-7030, doi:10.1523/JNEUROSCI.1954-08.2008 (2008).

9 Domingos, A. I. *et al.* Leptin regulates the reward value of nutrient. *Nature neuroscience* **14**, 1562-1568, doi:10.1038/nn.2977 (2011).

10 Domingos, A. I., Vaynshteyn, J., Sordillo, A. & Friedman, J. M. The reward value of sucrose in leptin-deficient obese mice. *Molecular metabolism* **3**, 73-80, doi:10.1016/j.molmet.2013.10.007 (2014).

11 Domingos, A. I. *et al.* Hypothalamic melanin concentrating hormone neurons communicate the nutrient value of sugar. *eLife* **2**, e01462, doi:10.7554/eLife.01462 (2013).

12 Tellez, L. A. *et al.* Separate circuitries encode the hedonic and nutritional values of sugar. *Nature neuroscience* **19**, 465-470, doi:10.1038/nn.4224 (2016).

13 Di Spiezio, A. *et al.* The LepR-mediated leptin transport across brain barriers controls food reward. *Molecular metabolism*, doi:10.1016/j.molmet.2017.12.001 (2017).
